# COVID-19–Considerations for the Female Athlete

**DOI:** 10.3389/fspor.2021.606799

**Published:** 2021-02-16

**Authors:** Georgie Bruinvels, Nathan A. Lewis, Richard C. Blagrove, Dawn Scott, Richard J. Simpson, Aaron L. Baggish, John P. Rogers, Kathryn E. Ackerman, Charles R. Pedlar

**Affiliations:** ^1^Faculty of Sport, Health and Applied Science, St Mary's University, Twickenham, United Kingdom; ^2^Orreco Ltd., Unit 103, Business Innovation Centre, NUI Galway, Galway, Ireland; ^3^School of Sport, Exercise and Health Sciences, Loughborough University, Loughborough, United Kingdom; ^4^School of Science and Health, University of Western Sydney, Penrith, NSW, Australia; ^5^Department of Nutritional Sciences, University of Arizona, Tucson, AZ, United States; ^6^Department of Pediatrics, University of Arizona, Tucson, AZ, United States; ^7^Department of Immunobiology, University of Arizona, Tucson, AZ, United States; ^8^Cardiovascular Performance Program, Massachusetts General Hospital, Boston, MA, United States; ^9^Department of Sports Medicine, Manchester Institute of High Performance, Manchester, United Kingdom; ^10^Manchester University NHS Foundation Trust, Manchester, United Kingdom; ^11^Boston Children's Hospital and Harvard Medical School, Boston, MA, United States

**Keywords:** athlete, coronavirus, return-to-play (return-to-sport), menstrual cycle, women

## Abstract

The SARS CoV-2 virus (COVID-19) caused the whole sporting calendar to be paused. As we embark on the challenge of navigating through the return to play (RTP) process, there is a necessity to consider the needs of all athletes. This commentary specifically considers recommendations and requirements for the female athlete with a physiological emphasis during and following the COVID-19 pandemic, however, it will be relevant for any similar future scenarios that may present. It is important to acknowledge that there remain many unknowns surrounding COVID-19 and the female athlete both in the short- and long-term.

## Background

As we continue to navigate through the SARS CoV-2 virus (COVID-19) pandemic, sports science and sports medicine colleagues are concerned with maximizing the safety of athletes in the return to play (RTP) phase and minimizing all short- and long-term health risks. The objective of this paper is to provide sports medicine and sports science practitioners RTP considerations specifically pertaining to the female athlete based on current research.

To date, the vast majority of sex-disaggregated data suggest that more men than women are dying from or experiencing more adverse outcomes as a result of COVID-19 infection (Griffith et al., 2020). A number of underlying behavioral, cultural, and biological causes have been proposed to explain this trend. Firstly, men are more likely to smoke (Liu et al., 2017), drink alcohol, be less vigilant with risk-reducing strategies such as handwashing, and have comorbidities (e.g., hypertension and diabetes) associated with a greater risk of a worsened outcome from COVID-19 (Gerdts and Regitz-Zagrosek, 2019; Mellström, 2020). Secondly, distinct immunological differences exist between men and women, expressed upon activation with a virus (Klein and Flanagan, 2016). This has, in part, been attributed to the X chromosome, which appears to have a role in innate and adaptive immunity (Conti and Younes, 2020). More specifically, genes encoded by the X chromosome are associated with a lower viral load and less inflammation. It has been suggested that sex hormones can also have divergent effects on the immune system; estrogen and progesterone may have immune-protective and anti-inflammatory properties (Sue, 2017). Menstruating women have greater numbers of CD4+ T cells, and typically produce more antibodies on viral infection when compared to men (Conti and Younes, 2020). The influence of sex also appears relevant in the context of viral entry of COVID-19 via the angiotensin-converting enzyme 2 (ACE-2) receptor (Li et al., 2020). The sex differences in the renin-angiotensin-aldosterone system could additionally have a role in the highlighted differences in infection severity. Although research is still in its early stages, the differences in sex hormones appear to modulate the expression of ACE-2 and transmembrane serine protease 2 (TMPRSS2), both of which are involved in determining viral entry and viral development of COVID-19 (Foresta et al., 2020; Li et al., 2020).

Recent research has suggested that infection rates in 10- to 50-year-old women are higher compared to men in the same age bracket, but lower in girls under the age of 10 and in women over the age of 50 when compared to boys and men of the same ages (Mauvais-Jarvis et al., 2020). This could be attributed to several factors, including cultural roles and gender norms such as work, childcare, social interactions, all of which are also likely to be influenced by geographical location. The potential role of the ovarian sex hormones here is interesting; the ovarian sex hormones have been hypothesized to have protective qualities against fatal infection, but given that ages 10–50 years represents the typical reproductive window, further research needs to explore how the sex hormones may alter viral susceptibility (Marina and Piemonti, 2020). The role sex hormones may play in infection rate and infection severity may be ascertained through studying these factors across the female lifecycle, as hormonal profiles are significantly different in prepubertal girls, reproductive-aged women, peri-menopausal and post-menopausal women. However, future research must account for the many external lifestyle, behavioral, and cultural factors that could confound findings.

Research to date suggests that typically young, fit, healthy female athletes without comorbidities are at lower risk of experiencing severe symptoms and a fatal outcome if infected with COVID-19 compared to their male counterparts (Wu and McGoogan, 2020). However, as our understanding of the virus and its effects are still nascent, both the potential short- and long-term effects of COVID-19 infection in addition to the impact that periods of “lockdown” have had on female athletes remain largely unknown. It is also essential to better understand transmission, particularly in those who may be more vulnerable, including older support staff and co-habiting family members of athletes.

Other emerging risk factors associated with the virus include ethnicity, vitamin D status, hyperandrogenism, and inflammation (Kyrou et al., 2020). Those with underlying conditions associated with any of these factors should be flagged as being higher risk, regardless of sex. Similarly, polycystic ovary syndrome warrants specific attention, as recently highlighted (Kyrou et al., 2020). This dysfunction affects 4–12% of the female population and is associated with all the aforementioned risk factors, however, any associated risk between COVID-19 and polycystic ovary syndrome remains speculative with no available data as yet. It is also important to consider that those with underlying medical conditions that require regular treatment are likely to be disproportionately affected by the COVID-19 lockdown. For example, those with menstrual dysfunctions such as endometriosis, who require regular treatment, may not have been able to access medical services, particularly in certain countries where lockdown restrictions may have been tougher (Alviggi et al., 2020). It is also important to appreciate that there may have been a reluctance to seek medical support on the athlete's part due to concern over infection.

While understanding the sex differences associated with risk of COVID-19 infection is essential, other impacts as a result of the enforced lockdown also need to be considered through a sex and gender lens, particularly, in the context of canceled or postponed major sporting events and leagues, and factors associated with lockdowns.

## Emerging From Lockdown: Return to Play and “The New Normal”

The sporting community has or is pushing for RTP in many countries, if they have not done so already, albeit in a state of “new normal” [e.g., with physical distancing measures and regular polymerase chain reaction (PCR) testing in place]. However, this is associated with substantial risk, particularly as social distancing measures are highly challenging or impossible to implement in many sports training and competition environments. Rapidly diagnosing cases of COVID-19 in athletes and staff and isolating them from the team environment is essential to avoid further disruption. Robust immuno-protective strategies and a specific focus on recovery processes appear necessary. In team sports, detraining of important physical qualities associated with performance, a reduction in competitive match fitness and sport-specific skill and contact training, together with a congested schedule once competitive sport is resumed, are likely to result in a higher incidence of injury (Table 1). In individual sports, the physical and psychological impact of the competitive environment can cause added stress, particularly when individuals have not been in these scenarios for a prolonged period of time. This could also increase injury risk and cause additional mental stress.

**Table 1:** Key points table for consideration on return to play in female athletes.

**While in isolation**	**Considerations**
COVID-19 infection, or presence of symptoms in line with COVID-19	Mild symptoms: undertake a 10-day period of rest with a gradual RTP, which is monitored throughout.
	Hospitalization: undertake a comprehensive biomarker and cardiac screening with a gradual RTP only when key markers are back to normal.
Exercise training and injury risk	Where the situation allows, attempt to maintain physical fitness. “Maintenance” maybe a more appropriate goal than “progression” to avoid placing excess stress on the immune system and reduce risk of injury during a period where sports medicine support is severely limited. Individualize training plans based upon previously identified strengths and weaknesses.
	Where relevant and possible, consider the specificity of the training stimulus.
	Telehealth should be utilized where possible to increase access to sports medical services and decrease risk of transmission.
Mental health	Make mental health resources available to athletes.
	Maintain regular communication with athletes.
Menstrual cycle monitoring	Tracking of cycles and symptoms; where significant abnormalities are seen, attempt to identify and manage causation, alongside modification of training.
General health and well-being	Place extra attention on following a varied and sufficient diet, meeting sleep requirements and optimizing recovery. Avoid a state of low energy availability.
**Return to play**	
Testing and monitoring in elite athletes	Conduct *regular* molecular (amplification e.g., PCR) testing. If antibody testing is better established, athletes with a negative molecular and a positive antibody test, provided they have no other signs or symptoms of viral disease, should be considered safe to resume training and social contact. Seronegative athletes should be monitored closely as they are most susceptible to infection. Athletes with a positive molecular test should be isolated and managed/monitored for illness severity.
	Daily monitoring of body temperature and symptoms (alongside menstrual cycle monitoring to account for natural physiologic changes) should be performed.
	Where possible, weekly monitoring of biomarkers, such as those of oxidative stress and inflammation should be done.
	Deploy cardiac screening as appropriate.
	Administer a routine battery of sport-specific physical fitness tests to ascertain the level of detraining and physical readiness in individual athletes.
	Conduct a health screen of athletes prior to RTP, capturing any alterations or concerns around sleep, menstrual cycle status, nutrition, and mental health.
Exercise training and injury risk	Where training was compromised, a musculoskeletal review should be undertaken to ascertain state of readiness to return to “typical” training.
	A graded re-implementation of sport-specific physical fitness training should be conducted to address deficits during the lockdown period. Use rated perceived exertion and wellness monitoring to gauge fatigue and training adaptation.
Mental health	Make mental health resources available to athletes and consider use of online platforms to increase accessibility.
Menstrual cycle monitoring	Continue tracking menstrual cycle and symptoms; where significant abnormalities are seen, attempt to identify and manage causation, alongside modification of training.
General health and well-being	Continue to focus on sleep and nutrition, with a priority on recovery, particularly in circumstances where scheduling is more congested. A state of low energy availability should be avoided. Nutritional support should be considered, particularly for those most vulnerable or with a history of an eating disorder or disordered eating. Sleep monitoring may also be considered, particularly in those identified to have experienced regular sleep disturbances. Remain vigilant with social distancing practices.

*RTP, return to play; PCR, polymerase chain reaction*.

## COVID-19 Testing and Symptom Tracking

Comprehensive strategies for regular nucleic acid amplification (molecular) for COVID-19 antigen are warranted, together with daily monitoring of COVID-19 symptoms. The molecular tests will identify athletes with an active COVID-19 infection, allowing appropriate interventions to be put in place to manage the course of illness and to prevent onward transmission. Future use of serological antibody tests could identify athletes who have previously been exposed to the virus, which serves two purposes. Firstly, it would help identify those who have not been exposed and are therefore susceptible to infection. Secondly, it would identify those athletes with neutralizing antibodies and potential immunity, which could, in the future be essential in terms of managing COVID-19 restrictions. Asymptomatic athletes presenting with a negative molecular test and a positive antibody test are unlikely to be contagious and may have protective immunity against COVID-19, although further data are needed. There is evidence to suggest that immunity and the immune response changes across the menstrual cycle (Oertelt-Prigione, 2012), and recent case studies have demonstrated potential inaccuracies of the antigen test based on menstrual cycle phase (Zheng et al., 2020). Two cases have been reported whereby women experienced positive PCR tests alongside COVID-19 symptoms during menstruation, but the symptoms disappeared post menstruation and subsequent PCR tests were negative. However, symptoms returned alongside a positive PCR test when both individuals started their next menstruation (Zheng et al., 2020). While clearly much more data are needed to better understand how ovarian hormones may affect the clinical course of COVID-19 infection, practitioners should be mindful of timing in the menstrual cycle when conducting testing.

Athletes commonly underreport symptoms, particularly when this may prevent or delay RTP training (McDonald et al., 2016); therefore, capturing biomarker data relating to general wellness and recovery, for example inflammation and redox homeostasis, could be advantageous. Indeed, it is noteworthy that increased oxidative stress has recently been reported in both injured and ill elite athletes when longitudinally monitored (Lewis et al., 2020). Where full recovery has not been accomplished prior to resumption of habitual training, the subsequent risk of overreaching (progressing to non-functional overreaching and overtraining), further illness, and musculoskeletal injury is increased. While daily molecular testing is not feasible for all sport participation, at a minimum, frequent monitoring of symptoms and temperature should be implemented, and COVID-19 secure bubbles should be created to reduce risk of an outbreak within a sports team or training environment. Those athletes reporting symptoms in line with COVID-19 should be instructed to self-isolate, and depending on the severity, training may need to be temporarily halted (Hull et al., 2020). Other markers such as resting heart rate and heart rate variability may also be useful for tracking recovery. In menstruating women, it is important to account for physiological changes in basal body temperature and resting heart rate when undertaking daily measurements because fluctuations in ovarian hormones influence these parameters (Tenan et al., 2014; Bull et al., 2019). There is also the potential for concurrent menstrual symptoms and COVID-19 symptoms (e.g., achiness, fatigue, nausea, and headaches) (Bruinvels et al., 2020; Centers for Disease Control and Centers for Disease Control Prevention, 2020). Tracking menstrual cycles and symptoms alongside temperature, heart rate, and potential COVID-19 symptoms would reduce risk of ambiguity.

## Cardiopulmonary

The systemic nature of COVID-19 emphasizes the need for comprehensive medical screening of athletes prior to RTP. Recommendations for screening of cardiac and respiratory health of athletes have been published with no differentiation between approaches for males and females (Baggish et al., 2020; Hull et al., 2020). Hull et al. (2020) recommend that those who have been hospitalized due to COVID-19 infection should undergo full cardiac screening prior to return to physical activity and those who had mild symptoms should undertake a prolonged rest period and conservative RTP strategy (10-day period of rest from onset of symptoms plus seven days after symptom resolution). While this strategy is recommended to minimize the possibility of viral myocarditis in athletes returning to full training too early (Hull et al., 2020), performing less strenuous activity during this recovery period is consistent with the guidelines published for the general population and has been recommended to promote anti-viral immunity and hasten viral resolution (Simpson and Katsanis, 2020).

Comprehensive cardiac screening should be performed in athletes who have been hospitalized with COVID-19. Failing to identify COVID-19-related heart scarring or latent myocarditis could result in an increase in exertional sudden cardiac death in the future, and we have an opportunity to study and prevent this upon reintroduction to sport across both sexes. Interestingly, some research suggests that the incidence and outcome of pneumonia and myocarditis can be more favorable in women vs. men with COVID-19 (Fairweather et al., 2013; Al-Baadani et al., 2019), however, there are currently too many unknowns for divergent sex-specific post-infection cardiac screening.

## Mental Health

During uncertain and life-changing times, such as the COVID-19 pandemic, increased susceptibly to undesirable states of anxiety and stress are likely (Weinberg and Cooper, 2012) therefore the impact of the COVID-19 pandemic on psychological and emotional health must also be considered. The 2003 SARS outbreak in Hong Kong was shown to have detrimental long-term consequences on mental health (Mak et al., 2009). Regardless of sex and gender, changes to competition scheduling, alterations to training regimens, reduced access to facilities and social isolation all have the potential to create a significant degree of uncertainty. A study evaluating the impact of COVID-19 on semi-elite and elite South African athletes found females to be more likely to report depressive feelings, energy loss and a lack of motivation when compared to male equivalents (Pillay et al., 2020). Similarly, other studies evaluating the effects of the recent COVID-19 period have found female athletes to be more likely to report higher scores of perceived stress and dysfunctional psychobiosocial states (*n* = 1137; di Fronso et al., 2020), and female footballers to be more likely to experience neuroticism and psychological inflexibility, while also having more concern about the impact of lockdown on sports performance (*n* = 175; Clemente-Suárez et al., 2020). There have been, and still are, ostensible geographical differences in the duration of and severity of lockdown, which is and likely to be reflected in access to facilities, sports medicine support and competition. Inevitability this “uneven playing field” has the potential to create significant anxiety for those competing internationally. Regardless, travel restrictions have been implemented globally to some degree and all international athletes are affected by changes to competition scheduling.

A survey completed by FIFPRO in March and April 2020 found almost twice the number of female soccer players exhibited depressive symptoms compared to men (FIFPRO, 2020). A recent systematic review also concluded that elite female athletes are more susceptible to anxiety than their male counterparts (Rice et al., 2019). It is therefore possible that the impact of isolation and other aspects of the pandemic on mental health is greater in women. This is of particular significance given the common lack of funding in women's sport. In many situations, women's sport is not the priority in terms of access to support services and training facilities, and concern has been raised in the mainstream media (Sports, 2020).

Women's sport is also under substantial financial strain. In some sports, professional athletes were furloughed (Sanders, 2020), and in others, the absence of competition has resulted in no or very limited income, clearly causing a significant degree of anxiety. While these issues are not exclusive to women, the likelihood is that more women will fit into this category. It is particularly important to consider that many of the industries in which job losses are common are dominated by women (e.g., hospitality, retail, tourism), therefore this may impact financial security, increasing levels of anxiety in female athletes (Ramos, 2020). Further, women are more often primary caregivers for children, making them more likely to have to compromise on their working status and training patterns (Ramos, 2020). Given the protective role that exercise can play in mental well-being, where possible, participation in physical activity should be consistently encouraged (Faulkner et al., 2020). The extent to which this is relevant is again likely to be highly influenced by the rules pertaining to specific geographical locations. Resources to support mental health should be made readily available, as highlighted in Table 1, with more comprehensive advice for practitioners outlined elsewhere (Reardon et al., 2020).

## Injury Risk/Facilities

Specialist attention needs to be given to ensuring that the resumption of training is carefully managed and progressed. For a variety of reasons, women are less likely to have access to specialist facilities and equipment to maintain their fitness during isolation (Bowes et al., 2020). Many women are also less likely than their male counterparts to have access to the specialist sports medicine support to facilitate recovery when competition is resumed. It is also well-established that sudden increases in load increase injury risk, while also compromising the immune system (Schwellnus et al., 2016). Therefore, when training has been compromised, a graded return to full training is warranted. In sports that have already been through the RTP process, a disproportionately large number of injuries have been observed; for example, it has been highlighted that an abnormally large frequency of anterior cruciate ligament (ACL) injuries occurred (12 ACL injuries) across the preseason and first five games (of a 22-game season) in the Swedish Women's League (Eriksson, 2020). An earlier study found 13 first-time ACL injuries across the entire 2012 season (Johnson et al., 2016). A study investigating sleep in early onset of lockdown in Spain found women to report a greater reduction in sleep quality compared to their male counterparts (Mon-López et al., 2020). Given the known relationship between sleep and injury risk, this risk factor should also be considered (Watson, 2017). This clearly highlights the need for extra consideration of sex and gender and the lockdown environment when evaluating requirements for RTP (Table 1). Sleep monitoring could be considered to support athletes through the RTP process.

Research in elite women's soccer, youth soccer, collegiate female soccer, lacrosse, and basketball, has shown an increase in non-contact injury prevalence either in the first month after preseason, or in the early part of the season (Jacobson and Tegner, 2007; Gall et al., 2008; Anderson et al., 2019). One study attributed this to the levels of physical and physiological fitness not being appropriate to withstand the intensity and frequency of competitive games. An increased risk of injury has also been shown in female athletes after a 2- to 3-week winter holiday break (Ruiz-Pérez et al., 2019). The present COVID-19 scenario has no historic parallel in male or female sport, but the 2011 National Football League (NFL) lockout that lasted nearly 19 weeks offers clues about the imminent risks of injury. Upon resumption of training and competition, 12 Achilles tendon ruptures occurred, 10 of which were in the first 12 days, and this was double the number expected based on historical averages across the whole season (Myer et al., 2011).

Epidemiological studies in women's soccer show that women are less likely to sustain an injury than men, however, where injuries are sustained, severity is greater and RTP is longer in women (Larruskain et al., 2018). Specific risk areas for the female athlete in team sports are ankle and knee injuries (Larruskain et al., 2018). Therefore, a focus on risk reduction strategies targeted at preventing these injuries is warranted during this period of isolation and during the early phase of RTP (Table 1). Increased psychological stress and negative life event stress are also associated with an increased risk of injury (Soligard et al., 2016), further delineating the importance of managing load and monitoring mental health where possible (Table 1).

Women's sports facilities and training areas are typically smaller, and budgets inferior to those in men's sports. This may make following best practice/government guidelines (i.e., social distancing and ensuring appropriate processes are followed to reduce risk of virus transmission) more challenging. Ideally, advanced planning should be used to facilitate as smooth a RTP as possible (Table 1). Telehealth should be considered as an alternative option, bridging the gap and increasing access while also minimizing risk of transmission.

## Menstrual Cycle Monitoring

The menstrual cycle is often termed a “vital sign,” providing an indication of overall health status (American Academy of Pediatrics Committee on Adolescence et al., 2006). A regular menstrual cycle is associated with an adaptive physiological state and provides a robust indication that the hypothalamic pituitary gonadal (HPG) axis is functioning correctly, and not exposed to excessive stress (Sokoloff et al., 2016). Examples of common stressors known to disrupt the HPG axis include inadequate fueling strategies, psychological stressors, excess physical stress or significant alterations to physical stress, travel, and sleep (Gollenberg et al., 2010; Iwasa et al., 2017). These stressors can result in a state of functional hypothalamic amenorrhea (FHA) or could increase the severity of symptoms. The COVID-19 pandemic has increased the likelihood of sleep disturbances, caused changes to exercise behaviors and negatively affected mental health (Bowes et al., 2020; Pillay et al., 2020; Ramos, 2020), with significant concern around the pandemic and COVID-19 infections both exacerbating existing eating disorders and increasing risk of disordered eating behaviors (Reardon et al., 2020; Touyz et al., 2020). In fact, 24.7% of elite Australian athletes have experienced menstrual cycle changes (McNamara et al., 2020) because of the pandemic. Again, geographical location should be considered here; it is plausible to suggest that disturbances could well be higher in countries where COVID-19 lockdowns have been more severe and numbers of fatalities higher.

Practitioners should therefore be mindful of the potential for changes to menstrual patterns, and where possible, monitor cycles and symptoms throughout the RTP process, using this as an indicator of readiness and overall wellness (Table 1). This is particularly relevant in light of the increased risk of illness and injury associated with FHA (Melin et al., 2019), and the positive association between inflammatory markers and menstrual symptoms (Bertone-Johnson et al., 2014), again potentially increasing susceptibility to illness and injury. Low energy availability in particular is associated with a delay in recovery from illness, injury, and general exercise training, thus it is important to avoid a calorie deficient state during this time (Melin et al., 2019).

Two recent studies found combined hormonal contraception users have higher blood concentrations of certain inflammatory markers (Cauci et al., 2017), and markers of oxidative stress compared to non-users (Kowalska and Milnerowicz, 2016). However, the impact this may have on viral activation and response is not known. Another study demonstrated viral immunosuppression in cell cultures from women using medroxyprogesterone, but not other forms of progestin (Huijbregts et al., 2014). Although it is clear that more research is needed here, it would be advisable to take extra care when monitoring training load and wellness in those using hormonal contraception.

## Conclusions

While the threat of COVID-19 remains, even during the rollout of widespread vaccination, applying a physiological lens, it is essential to undertake daily monitoring of athletes to capture any symptoms that are consistent with COVID-19 infection, including raised body temperature. Symptom screening is a minimum precaution to protect athletes, support staff and their families and to prevent further competition- and league-wide competition shutdowns. Frequent monitoring of resting heart rate, biomarkers of inflammation and oxidative stress, where possible, and menstrual cycle tracking, would further augment female athlete care while sports adapt to a new normal and the influence of COVID-19 gradually subsides. Historically, there has been a lack of inclusion and consideration of female-specific needs in scenarios (Mantovani et al., 2020; Wenham et al., 2020), but there is an opportunity to use this scenario as a chance to provide essential parity in athlete care, with attention to sex and gender differences, across sports as they emerge from this unprecedented hibernation. Where resources are limited, practitioners should be directed to undertake comprehensive medical evaluations of athletes who have suffered significant symptoms or hospitalization.

## Author Contributions

All authors contributed to and reviewed the final draft of this manuscript.

## Conflict of Interest

GB, NL, and CP are consultants or are employed with Orreco, creators of the FitrWoman app. The remaining authors declare that the research was conducted in the absence of any commercial or financial relationships that could be construed as a potential conflict of interest.
